# Sex Differences in the Effect of Resveratrol on DSS-Induced Colitis in Mice

**DOI:** 10.1155/2017/8051870

**Published:** 2017-03-29

**Authors:** Alexandra Wagnerova, Janka Babickova, Robert Liptak, Barbora Vlkova, Peter Celec, Roman Gardlik

**Affiliations:** ^1^Faculty of Medicine, Institute of Molecular Biomedicine, Comenius University, 811 08 Bratislava, Slovakia; ^2^Faculty of Medicine, Institute of Medical Physics, Biophysics, Informatics and Telemedicine, Comenius University, 811 08 Bratislava, Slovakia; ^3^Faculty of Medicine, Institute of Pathophysiology, Comenius University, 811 08 Bratislava, Slovakia; ^4^Faculty of Natural Sciences, Department of Molecular Biology, Comenius University, 842 15 Bratislava, Slovakia

## Abstract

Resveratrol is a natural polyphenol studied for its possible protective properties in inflammatory bowel diseases. Moreover, it has been shown to interact with estrogen receptors. In the present study, we aimed to investigate possible diverse effects of resveratrol on female and male mice in DSS-induced colitis. Thirty-seven C57BL/6 mice (21 female and 16 male) were divided into three groups for each sex. The first group received pure water (CTRL). The other two groups received 1.5% dextran sulfate sodium (DSS) to induce colitis from which one group was treated with resveratrol (DSS + RSV). Intake of 1.5% DSS caused weight loss in all DSS groups compared to control mice. Weight loss, stool consistency, and discomfort did not show any protective effect of resveratrol in males and showed even adverse effects in females. In females, the activity of myeloperoxidase was lower compared to that in males. However, colon length and spleen weight showed no sex differences, which can indicate the induction of only mild colitis in mice. Resveratrol did not have any effect on TNF-alpha levels. Taken together, these results for the first time propose possible diverse effects of resveratrol in DSS-induced colitis model depending on the sex of the animal. However, this conclusion must be confirmed by further analyses.

## 1. Introduction

Inflammatory bowel disease (IBD) including ulcerative colitis and Crohn's disease (CD) is characterized by uncontrolled inflammation of the intestinal mucosa [1]. Mechanisms involved in pathogenesis still require a better understanding, and a need for new therapeutic strategies is rising [2–4].

Resveratrol (3,4′,5-trihydroxy-trans-stilbene; RSV) is a natural polyphenol found in various plants, grapes, peanuts, or red wine [5]. Resveratrol is widely studied for its broad range of biologic properties including antioxidant, antitumor, anti-inflammatory, cytoprotective, antimutagenic, proapoptotic, and immunoregulatory activities [6–10]. Its protective abilities have been shown in cardiovascular, neuronal, renal, or hepatic diseases but also in diabetes [5, 11–14]. Based on resveratrol's anti-inflammatory nature, recent literature suggests its beneficial effect in various animal models of colitis. For example, in dextran sulfate sodium- (DSS-) induced colitis in mice, both oral and dietary administration of resveratrol caused reductions of proinflammatory cytokines, TNF-*α*, IFN-*γ*, IL-8, and IL-1*β*, and an increase of the anti-inflammatory cytokine IL-10. Moreover, it reduced PGES-1, COX-2, and iNOS protein expressions and increased tissue SOD and GSH-Px activities involved also in oxidative stress progression [15, 16]. In addition, it has been shown that resveratrol may protect against colitis through upregulation of SIRT1 in immune cells in the colon and is closely associated with the regulation of Treg/Th17 balance and the HIF-1*α*/mTOR signaling pathway [17, 18]. In another mouse colitis model using IL-10^−/−^ mice which develop spontaneous chronic colitis within 18 weeks, resveratrol was able to induce immunosuppressive CD11b^+^ Gr-1^+^ MDSCs in the colon and suggests an as-yet-unidentified mode of anti-inflammatory action of this plant polyphenol [19]. Similar positive outcomes of resveratrol treatment were demonstrated in various rat colitis and Crohn's disease models including 2,4,6-trinitrobenzene sulfonic acid (TNBS), oxazolone, and peptidoglycan-polysaccharide [20–23]. Furthermore, Samsami-Kor et al. performed a pilot study on patients with ulcerative colitis showing that 6 weeks of resveratrol supplementation was able to improve quality of life in patients with active colitis at least partially through inflammation reduction. However, further studies with longer duration are needed to confirm the clinical application of resveratrol [24].

Considering the structural similarities between resveratrol and diethylstilbestrol, a synthetic estrogen, resveratrol has been shown to interact with estrogen receptors (ER) [6] and has thus been designated as a “phytoestrogen” [25, 26]. A number of resveratrol-sensitive tissues are ER-positive. However, the two ER subtypes found in mammals, ER*α* and ER*β*, show different biochemical properties and tissue distribution [27, 28]. Bowers et al. reported that those tissues that uniquely express ER*β* or that express higher levels of ER*β* than ER*α* may be more sensitive to resveratrol's estrogen agonist activity and that resveratrol's antagonist activity was only observed with ER*α* [29]. Moreover, ER activates transcription in response to estradiol, and Bhat et al. showed that in the absence of estrogen, resveratrol exerts mixed estrogen agonist/antagonist activities in some mammary cancer cell lines, but in the presence of estrogen, resveratrol functions as an antiestrogen [25]. Further investigation also revealed that the anti-inflammatory response of resveratrol depends on its pathway-selective ability as ER*α* ligand. This binding changes the shape of the receptor in a way that controls which coregulator molecules help it to regulate transcription [30].

In the present study, we aimed to investigate possible diverse effects of resveratrol on female and male mice in DSS-induced colitis.

## 2. Material and Methods

### 2.1. Animals

Fifty C57BL/6 mice (25 female and 25 male) at age of 14 weeks were obtained from Charles River Laboratories (Prague, Czech Republic). Mice were kept in mixed gender housing in standard lab cages with stainless steel wire tops and standard bedding and in a controlled environment with 12 : 12 light-dark cycle with ad libitum access to water and feed. For the experiment, animals were divided into three groups, 5 animals per cage: female—CTRL (*n* = 5), DSS (*n* = 10), and DSS + RSV (*n* = 10); male—CTRL (*n* = 5), DSS (*n* = 10), and DSS + RSV (*n* = 10). For the final analysis, only mice which survived the experiment were included: female—CTRL (*n* = 4), DSS (*n* = 8), and DSS + RSV (*n* = 9); male—CTRL (*n* = 4), DSS (*n* = 7), and DSS + RSV (*n* = 5).

### 2.2. Colitis Model and Resveratrol Treatment

To induce colitis, the DSS and DSS + RSV groups received 1.5% DSS (molecular weight (MW) 36,000–50,000; no. 160110; MP Biomedicals, Solon, OH, USA) for 12 days ad libitum in drinking water starting from day 0. The CTRL group received water ad libitum. Fresh DSS solution and water were prepared every 3 days. From day 12, DSS was changed back to water for one day. Resveratrol (Cat. number J60790, CAS number 501-36-0, Alfa Aesar, Germany) was dissolved in 50% ethanol and administered to the DSS + RSV group using oral gavage at concentration 100 mg/kg of mouse per day from day 0 through 11 days. Other groups received 50% ethanol. Mice were sacrificed on day 13. The animal experiment was approved by the Institutional Review Board and Ethics Committee of Comenius University, Faculty of Medicine.

### 2.3. Daily Observations and Scoring

Body weight, stool consistency, and discomfort were monitored on a daily basis starting from day 0 until the end of the experiment. Weight loss was expressed as a percentage of the initial weight of the animal. Stool consistency scoring: 0, normal; 1, soft formed; 2, watery; and 3, watery with blood [31, 32]. Discomfort consisted of three parameters: appearance (0, normal; 1, mild piloerection; 2, moderate piloerection, mild hunched back; and 3, severe piloerection, hunched back), behavior (0, normal; 1, mildly reduced activity; 2, severely reduced activity; and 3, no activity), and reactivity (0, normal; 1, moves when stimulated; 2, reluctant to move; and 3, no movement). An overall animal discomfort score was calculated as average score from added up scores for appearance, behavior, and reactivity per time point per experimental group [33].

### 2.4. Collection of Samples

Mice were anesthetized and the entire colon was removed from the cecum to the anus. The length of the colon was measured, then washed with phosphate buffer solution to remove the remaining content, and weighed. Spleen was removed and weighed. Samples from the colon were taken, divided into 0.5 cm long pieces, snap frozen in liquid nitrogen, and stored at −80°C until use.

### 2.5. Biochemical Analyses

Samples taken from distal and proximal colon tissue were homogenized using TissueLyser II (Qiagen, Hilden, Germany). Following the centrifugation of distal colon homogenates, the supernatants were used for measurement of myeloperoxidase (MPO) levels using a protocol described by Kim et al. [34]. Tumor necrosis factor alpha (TNF-alpha) was measured in proximal colon homogenates using Mouse TNF-alpha Quantikine ELISA Kit (Cat. number: MTA00B, R&D Systems, Inc., MN, USA). The concentration of proteins in the colon homogenates was analyzed using the Lowry assay [35], and cytokine concentrations were expressed in U/mg of proteins (MPO) or pg/mg proteins (TNF-alpha).

### 2.6. Statistical Analysis

Data were analyzed using Student's *t*-test, one-way, and two-way analysis of variance. *p* < 0.05 was considered statistically significant. The calculations were performed with GraphPad Prism 5 software (GraphPad Software, San Diego, CA, USA). Data were presented as mean ± SD. Additional statistics for two-way analysis of variance (*F* values, *t* values) is provided in Supplementary Material available online at https://doi.org/10.1155/2017/8051870.

## 3. Results

### 3.1. Effect of DSS and Resveratrol on Weight Loss

**Figure 1:**
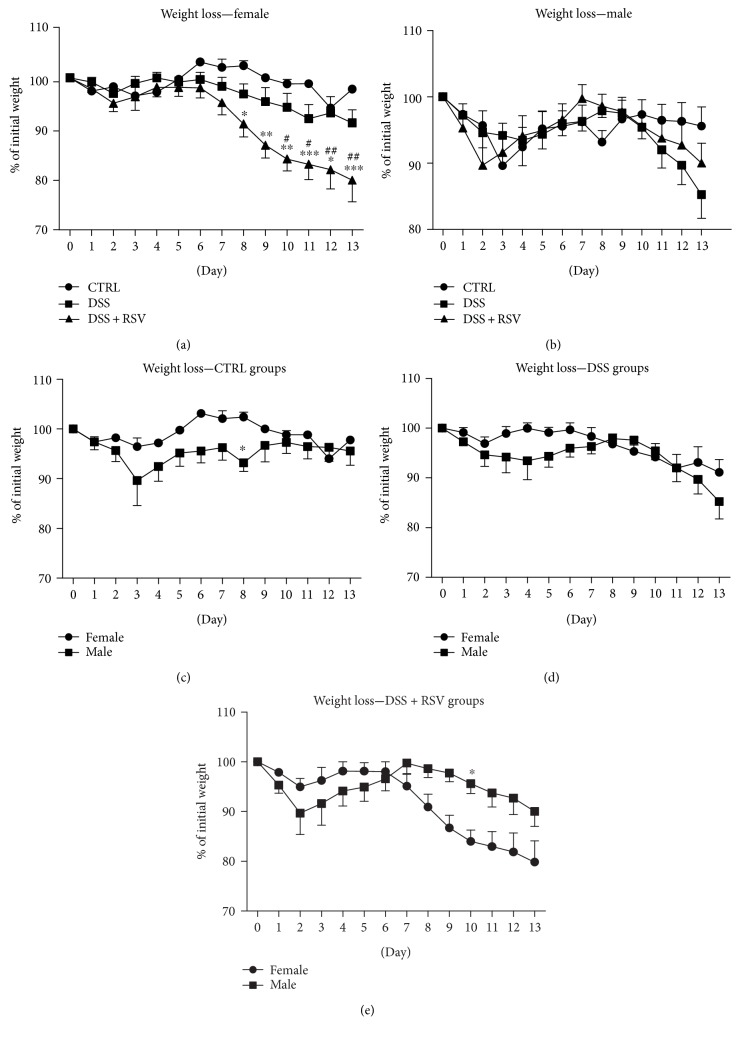
Weight loss time course. In females, significant weight loss was observed in the DSS + RSV group compared with that in the CTRL from day 8 and in the DSS + RSV group when compared with that in the DSS group from day 10. ^∗^*p* < 0.05, ^∗∗^*p* < 0.01, and ^∗∗∗^*p* < 0.001, in comparison with the control; ^#^*p* < 0.05 and ^##^*p* < 0.01, DSS + RSV in comparison with DSS. In males, there was no significant difference between the groups. Significant differences between sexes were observed in the CTRL groups on day 8 and in the DSS + RSV on day 10. ^∗^*p* < 0.05, males in comparison with females. CTRL, control; DSS, dextran sulfate sodium; RSV, resveratrol.

In females, intake of 1.5% DSS caused significant weight loss in days 8, 9, 10, 11, 12, and 13 (*p* < 0.05, *p* < 0.01, *p* < 0.01, *p* < 0.001, *p* < 0.05, and *p* < 0.001, resp.) in the group treated with RSV when compared with that in control (Figure 1). Significant weight loss from day 10 was observed in the DSS + RSV group compared with the DSS group (*p* < 0.05, *p* < 0.05, *p* < 0.01, and *p* < 0.01, resp.). There was no significant difference in the DSS group compared to that in the control. In males, there was no significant difference between the groups. Comparison of females and males showed significant weight difference in the CTRL groups on day 8 (*p* < 0.05) and in the DSS + RSV groups on day 10 (*p* < 0.05).

### 3.2. Effect of DSS and Resveratrol on Stool Consistency

**Figure 2:**
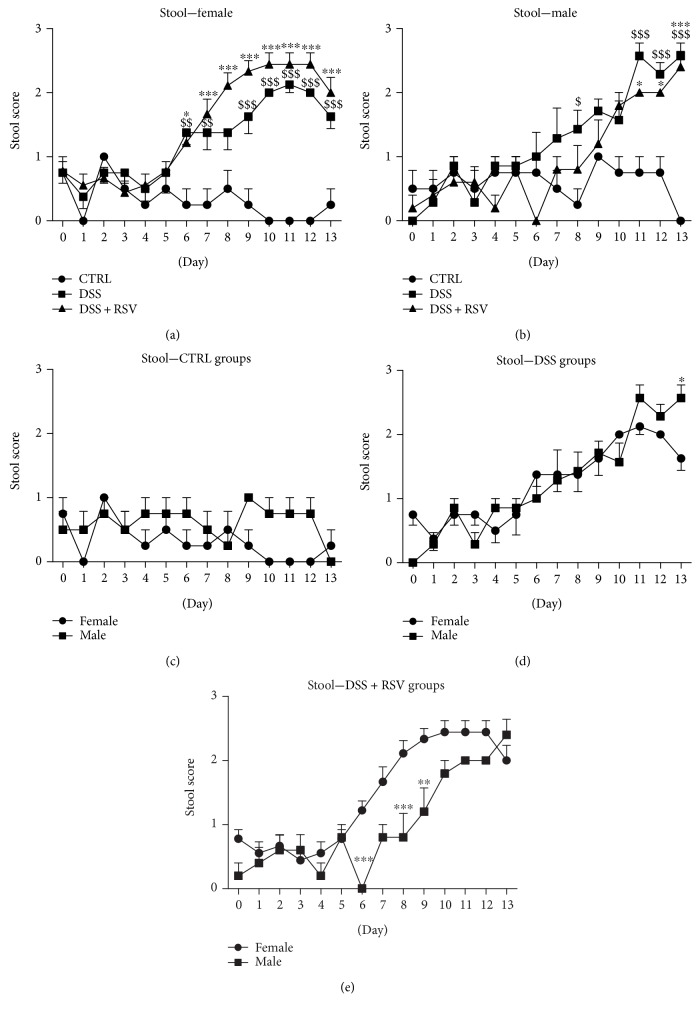
Stool consistency time course. In females, significantly higher score in stool consistency was observed in the DSS and DSS + RSV groups from day 6 when compared with that in the control. ^$$^*p* < 0.01 and ^$$$^*p* < 0.001; DSS in comparison with control; ^∗^*p* < 0.05 and ^∗∗∗^*p* < 0.001; 5DSS + RSV in comparison with control. In males, significantly higher score in stool consistency was observed in the DSS and DSS + RSV groups from day 11 when compared with that in the control. ^$$$^*p* < 0.001, DSS in comparison with control; ^∗^*p* < 0.05 and ^∗∗∗^*p* < 0.001, DSS + RSV in comparison with the control. Significant differences between sexes were observed in the DSS groups on day 13 and in the DSS + RSV groups on days 6, 8, and 9. ^∗^*p* < 0.05, ^∗∗^*p* < 0.01, and ^∗∗∗^*p* < 0.001, males in comparison with females. CTRL, control; DSS, dextran sulfate sodium; RSV, resveratrol.

In females, daily stool consistency observation showed development of inflammation with significant differences on days 6, 7, and 9 till 13 (*p* < 0.01 on days 6 and 7 and *p* < 0.001 from day 9 to day 13) in the DSS group when compared with that in the CTRL (Figure 2). Significant differences were also observed from day 6 (*p* < 0.05 on day 6 and *p* < 0.001 from day 7 to day 13) in the DSS group treated with RSV. In males, significant continuous inflammation was observed from day 11 in DSS (*p* < 0.001) and DSS + RSV (*p* < 0.05 on days 11 and 12, *p* < 0.001 on day 13) groups compared with that in CTRL. No significant differences between DSS and DSS + RSV groups were observed in any sex. Significant differences between sexes were observed in groups receiving DSS on day 13 (*p* < 0.05) and in groups receiving DSS in combination with RSV on days 6, 8, and 9 (*p* < 0.001, *p* < 0.001, and *p* < 0.01, resp.).

### 3.3. Effect of DSS and Resveratrol on Animal Discomfort

**Figure 3:**
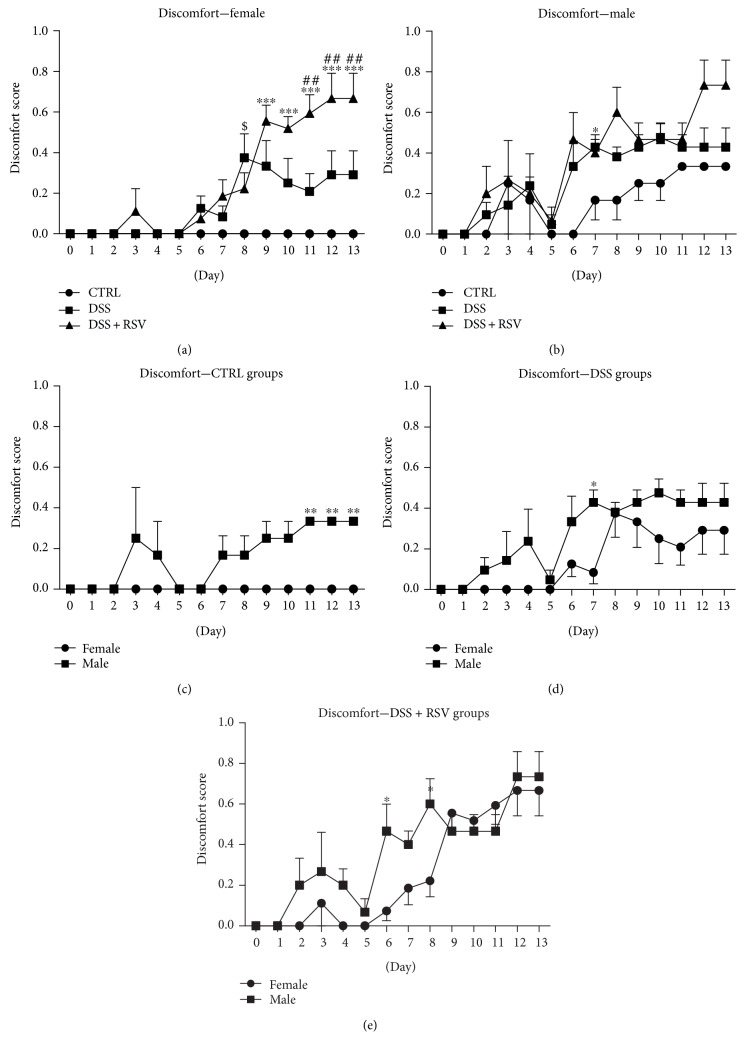
Discomfort time course. In females, a significantly worse score in discomfort was observed in the DSS + RSV group from day 9 when compared with that in control and from day 11 when compared with that in the DSS group. ^∗∗∗^*p* < 0.001, DSS + RSV in comparison with control; ^##^*p* < 0.01, DSS + RSV in comparison with DSS. Significant differences between sexes were observed in CTRL groups from day 11. ^∗∗^*p* < 0.01, males in comparison with females. CTRL, control; DSS, dextran sulfate sodium; RSV, resveratrol.

To monitor animal wellbeing, scoring of discomfort was performed. In females, significant differences were observed from day 9 to day13 in the DSS group treated with RSV compared with that in the control (*p* < 0.001 each day) (Figure 3). Significantly worse wellbeing was seen from day 11 in the DSS + RSV group when compared with the DSS group (*p* < 0.01). In males, there was no eminent difference between the groups. Comparison of females and males showed a significant difference in the CTRL groups on days 11 till 13 (*p* < 0.01 each day), in DSS groups on day 7 (*p* < 0.05), and in DSS + RSV groups on days 6 and 8 (*p* < 0.05 and *p* < 0.05, resp.).

### 3.4. Effect of DSS and Resveratrol on Colon Length

**Figure 4:**
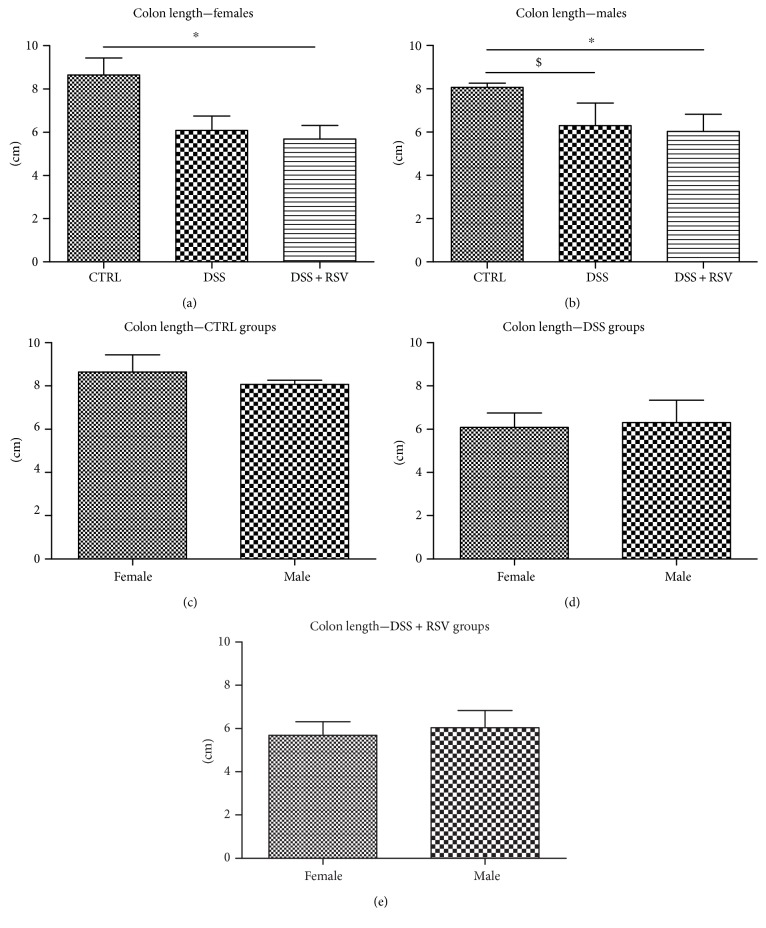
Colon length. In females, colon length was significantly shorter in the DSS + RSV group when compared with that in the control. ^∗^*p* < 0.05, in comparison with the control. In males, colon length was significantly shorter in the DSS and DSS + RSV groups when compared with that in the control. ^∗^*p* < 0.05, DSS + RSV in comparison with the control; ^$^*p* < 0.05, DSS in comparison with the control. There were no significant differences between sexes. CTRL, control; DSS, dextran sulfate sodium; RSV, resveratrol.

During colitis, inflammation processes are linked to colon shortening. In females, the length of the colon was significantly shortened in the DSS + RSV group compared with that in control (*p* < 0.05) (Figure 4). There were no significant differences between the DSS and control groups; however, the trend of shortening was observed. In males, significant colon shortening was observed in the DSS group (*p* < 0.05) as well as in the DSS + RSV group (*p* < 0.05) when compared with that in the control. There were no differences in any group between the sexes.

### 3.5. Effect of DSS and Resveratrol on Spleen Weight

**Figure 5:**
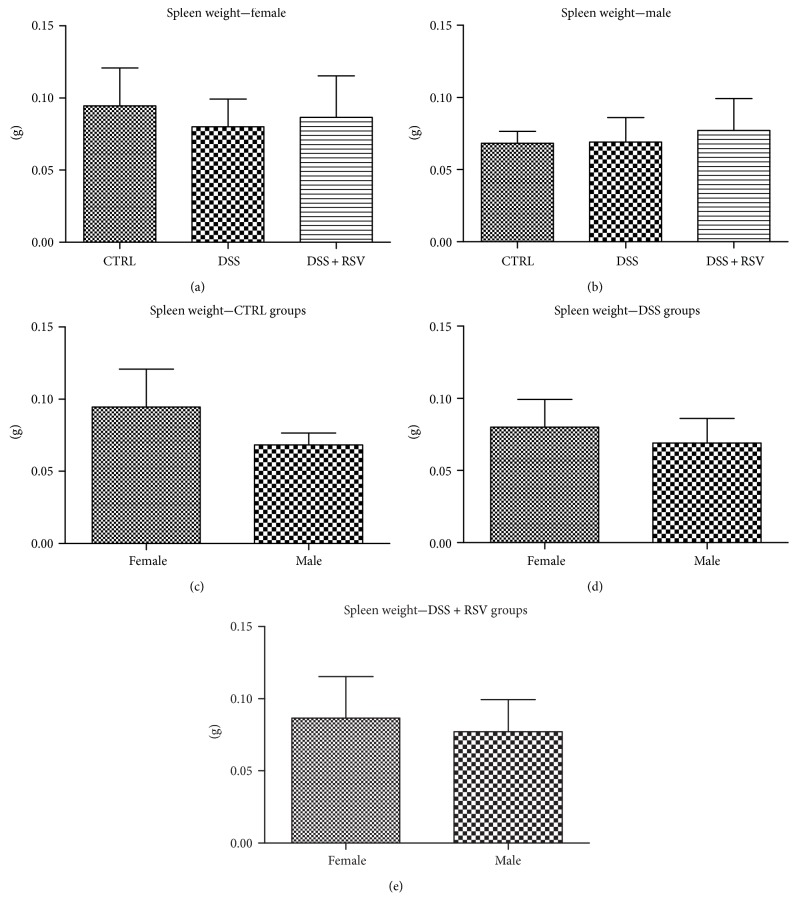
Spleen weight. In females and males, there were no significant differences in the DSS and DSS + RSV groups when compared with that in the control. There were no significant differences between sexes. CTRL, control; DSS, dextran sulfate sodium; RSV, resveratrol.

The last macroscopically measured parameter was the spleen weight as its enlargements are an accompaniment to DSS-induced colitis in mice. There was no difference in spleen weight in any of the groups receiving DSS when compared with the control (Figure 5). This was observed in both sexes. There were no differences in any group between the sexes.

### 3.6. Effect of DSS and Resveratrol on the Activity of Myeloperoxidase

**Figure 6:**
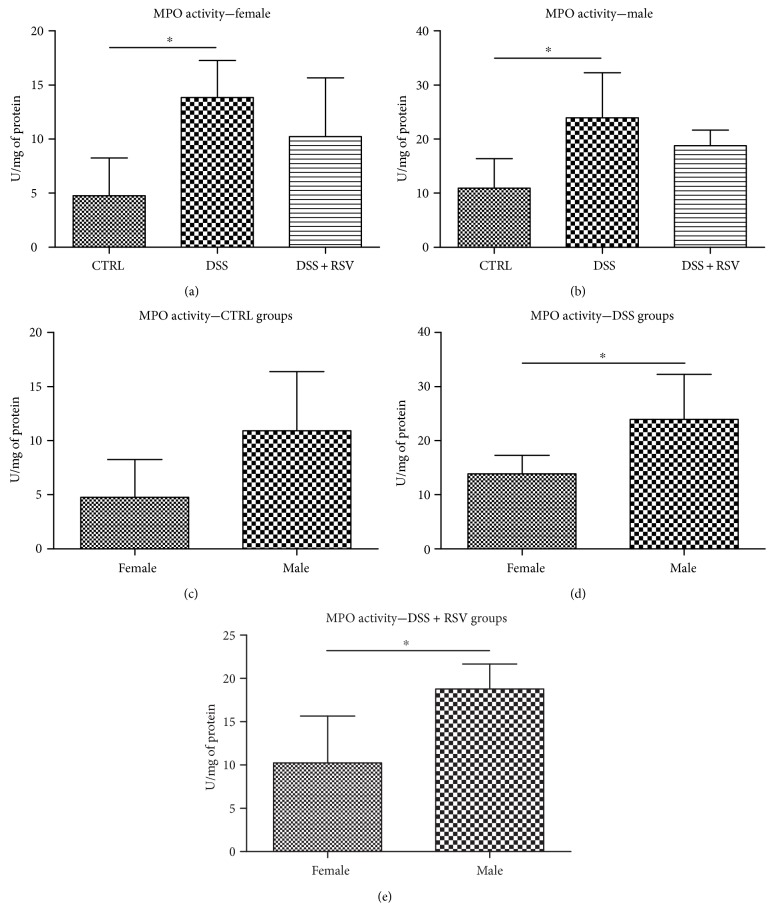
Myeloperoxidase quantification in colon homogenates. In females and males, MPO activity was significantly higher in the DSS groups in comparison with that in the control. ^∗^*p* < 0.05, in comparison with the control. In males, MPO activity was significantly higher in the DSS and DSS + RSV groups when compared with that in females. ^∗^*p* < 0.05, males in comparison with females. CTRL, control; DSS, dextran sulfate sodium; RSV, resveratrol.

Activity of MPO was measured in distal colon homogenates. In females as well as in males, there was a significantly higher MPO activity in groups treated with DSS when compared with that in control (*p* < 0.05 and *p* < 0.05, resp.) (Figure 6). In both sexes, in groups treated with DSS in combination with RSV, lowering of DSS activity was observed when compared with DSS groups, however not statistically significant. Differences between sexes were observed in groups DSS and DSS + RSV, where males had significantly higher MPO activity than females (*p* < 0.05 and *p* < 0.05, resp.).

### 3.7. Effect of DSS and Resveratrol on TNF-Alpha Concentration

**Figure 7:**
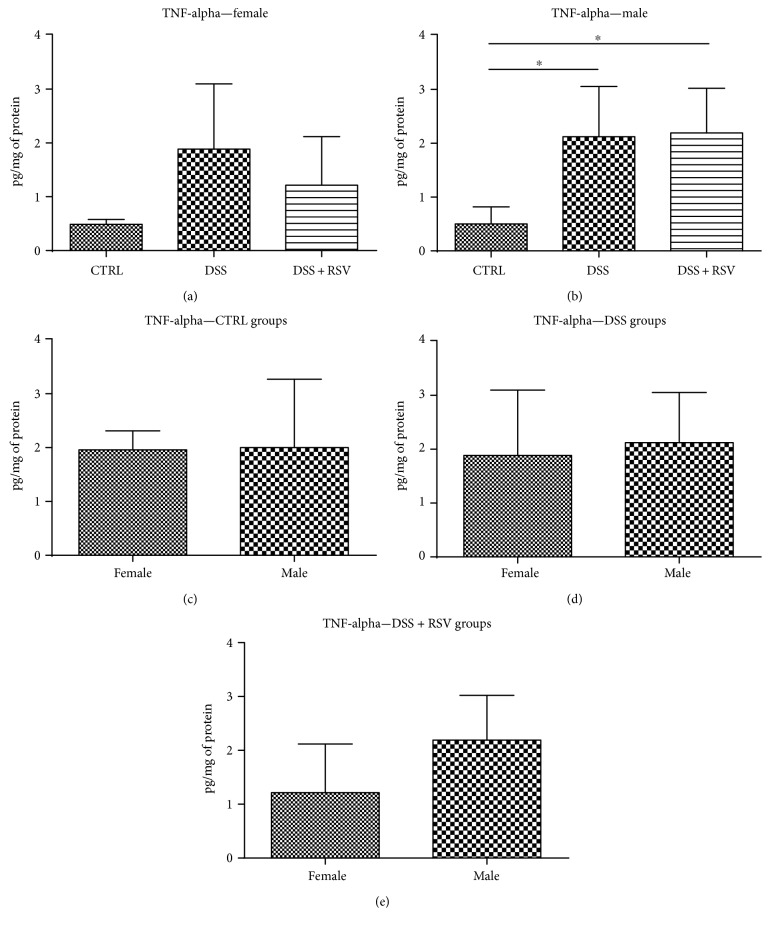
TNF-alpha quantification in colon homogenates. In males, TNF-alpha concentration was significantly higher in the DSS and DSS + RSV groups in comparison with that in the control. ^∗^*p* < 0.05, in comparison with the control. No significant difference between groups was found in females. Similarly, no significant difference between sexes was found in either of the groups. CTRL, control; DSS, dextran sulfate sodium; RSV, resveratrol.

TNF-alpha concentration was measured in proximal colon homogenates. In males, significantly higher TNF-alpha concentration was found in groups treated with DSS when compared with that in control (*p* < 0.05) (Figure 7). RSV groups did not show reduced TNF-alpha levels. However, there is a trend towards reducing TNF-alpha in colitic females treated with resveratrol. No sex differences were observed in either of the groups.

## 4. Discussion

From previous studies, it is known that resveratrol might possess some positive effects on the course of animal models of colitis [15, 16, 36, 37]. At the same time, it was found that resveratrol can act as an estrogen agonist or antagonist. Based on this, it can be hypothesized that resveratrol can have different effects depending on the sex of the animal. For example, Soylemez et al. showed that long-term administration of resveratrol increased vasorelaxation of the aorta, stronger in males than in females, while they theorized that it can be mediated through estrogen receptors [38]. Moreover, estrogen has been shown to affect certain cytochrome P450 isoenzyme concentrations in the liver [39]. Zendulka et al. confirmed the diverse effects of resveratrol depending on the sex of rats, where resveratrol acted as an inhibitor of cytochrome P450 2D2 and had a higher impact in males than in females [40]. Estrogen receptors together with estradiol are known to be able to regulate inflammatory paths of innate immune response which is disrupted during IBD [41, 42]. Based on these findings, we were interested in the effect of resveratrol in animal models of colitis with regard to the sex, which has so far not been investigated.

In contrast to the previous studies, weight loss and stool consistency did not show any protective effect of resveratrol in diseased females nor males. However, males showed no difference between resveratrol-treated and resveratrol-untreated colitis groups, and in females, administration of resveratrol even increased the weight loss. Also, the onset of diarrhea appeared earlier in females than in males. Adverse effect of resveratrol in colitis females was also addressed by monitoring of discomfort. The group treated with both DSS and resveratrol bore the experiment even worse than the group treated only with DSS where no statistically significant differences between DSS and control groups were observed; only a trend could be seen. Moreover, similar to previous results, in males, there were no differences between the colitis groups. All these results suggest possible diverse effects of resveratrol depending on the sex of the animal. Interestingly, comparing sexes of the control groups showed worse discomfort score in males, which might be due to a more sensitive reaction to oral gavage of males compared with females. However, this difference could not be seen in groups treated with DSS or DSS in combination with RSV. This could be influenced, especially in later time points, by starting colitis which also affected the wellbeing of mice in both sexes and could potentially dominate over the negative effect of gavaging. Worse toleration of oral gavage by males could also affect the mortality rate which was higher compared to that by females. From a total of 25 mice from each sex, 4 females and 9 males died, most of them at the beginning or in the middle of the experiment, when the colitis was still not fully developed (data not shown).

Colon length measurement showed similar results; however, there were no differences between the sexes. Based on these results, together with the spleen weight measurement, there were no differences between the groups nor the sexes; it can be assumed that we managed to induce only mild colitis in mice.

Interesting results showed the analysis of MPO activity. It indicates the presence of a higher number of granulocytes in the intestine of colitic males compared to females. Nevertheless, this is consistent with previous studies, while it can be theorized that testosterone might also influence the activity of neutrophils, which are the main MPO producers [43–45].

Mice with colitis had higher TNF-alpha levels compared to controls. In males, the resveratrol group showed no improvement compared to that in the DSS group. Unlike males, females treated with resveratrol showed some trend towards reduced TNF-alpha levels. This, however, was not significant. No clear sex differences were observed.

Despite the many nonsignificant data, the results indicate possible differences between the sexes, and based on the weight loss and stool consistency, it can be assumed a milder course of colitis is present in males than in females when treated with resveratrol. In our previous study, we showed that during DSS-induced colitis, female mice have milder course of disease presumably mediated by estradiol [43]. We attribute the opposite results of the current study to the ability of resveratrol to interact with estrogen receptors and especially its feature as possible estrogen inhibitor [25, 29]. On the other hand, the nature of the resveratrol plays an important role in the result inconsistency, especially when compared with the available literature. It was not possible to dissolve it in a lower concentration of ethanol, 50% of which might have negative effect on the mice physical state. Moreover, daily oral gavage can be beside increased stress accompanied by esophageal reflux, aspiration pneumonia, and esophageal and gastric rupture. These factors could affect both drink and food intake and thereby change the conditions for routine testing of colitis models. Daily drink and food consumption was variable during the experiment and showed no significant differences between the groups (data not shown). Therefore, in the future, we will need to solve the solubility of resveratrol, as well as modify the model so that the colitis can be induced in the shortest possible time, thereby reducing the number of necessary gavages.

Nevertheless, to our knowledge, these data represent the first attempt to assess the diverse effect of resveratrol depending on the sex in the course of DSS-induced colitis. Therefore, to confirm these results, more data will be needed and the colon histology as well as the expression of estrogen receptors has to be assessed.

## 5. Conclusions

When addressing a new therapeutic with known sex hormone-like effects, naturally, a question of its diverse action determined by sex arises. In this study, for the first time, we tried to answer this question for a widely studied resveratrol in connection with inflammatory bowel disease. As evident by myeloperoxidase levels, resveratrol does not have the possibility to decrease inflammation neither in females nor in males. As long as weight loss, stool consistency, and animal discomfort is concerned, resveratrol even worsens the ongoing colitis in females. This, however, is not the case in males. Thus, this study not only highlights the importance for deeper analysis of resveratrol's sex-dependent influence and interaction with hormones and their receptors but also highlights a cautiousness of the possibly sex-dependent beneficial or harmful effects of certain new therapeutics and food compounds, especially those that share some characteristics with hormones and thus might interfere with their pathways. The study is also relevant from the experimental design point of view, as it shows that the same administration procedure (gavaging) might cause worse discomfort for male compared to female animals. Therefore, when an experiment is arranged, all factors in addition to the treatment which could affect the outcome should be taken into account.

## Conflicts of Interest

The authors declare that they have no conflicts of interest.

## Supplementary Material

Supplementary statistics for 2-way ANOVA
