# Supplementary material for: Sex Differences in the Effect of Resveratrol on DSS-Induced Colitis in Mice
Source: Gastroenterol Res Pract. 2017 Mar 29;2017:8051870. doi: 10.1155/2017/8051870 (PMC5390549; doi:10.1155/2017/8051870)
Supplement: Supplementary file 1 — Supplementary statistics for 2-way ANOVA [file 8051870.f1.pdf]

Supplementary statistics for 2-way ANOVA

Figure 1. Weight loss time course. In females, there was a significant difference between CTRL and DSS+RSV groups at days 8 (t = 3.11), 9 (t = 3.61), 10 (t = 4.02), 11 (t = 4.31), 12 (t = 3.29), 13 (t = 4.86) and between DSS and DSS+RSV groups at days 10 (t = 3.43), 11 (t = 3.01), 12 (t = 3.75), 13 (t = 3.78) (a). Significant differences between sexes were observed in CTRL groups on day 8 (t = 3.25) and in DSS+RSV groups on day 10 (t = 2.96), males in comparison with females (c and e). CTRL, control; DSS, dextran sulfate sodium; RSV, resveratrol.

Variation showed these results:

**F values**	**a**	**b**	**c**	**d**	**e**
Interaction	4.30	1.51	3.74	1.35	7.15
Time	14.3	5.57	5.19	6.84	11.9
Column Factor	4.40	0.0661	2.21	1.22	1.14
Subjects (matching)	15.9	14.3	27.7	9.48	20.0

Figure 2. Stool consistency time course. In females, there was a significant difference between CTRL and DSS groups at days 6 (t = 3.47), 7 (t = 3.47), 9 (t = 4.24), 10 (t = 6.17), 11 (t = 6.56), 12 (t = 6.17), 13 (t = 4.24) and between CTRL and DSS+RSV groups at days 6 (t = 3.06), 7 (t = 4.46), 8 (t = 5.07), 9 (t = 6.55), 10 (t = 7.69), 11 (t = 7.69), 12 (t = 7.69), 13 (t = 5.51) (a). In males, there was a significant difference between CTRL and DSS groups at days 8 (t = 3.30), 11 (t = 5.11), 12 (t = 4.30), 13 (t = 7.21), between CTRL and DSS+RSV groups at days 11 (t = 3.27), 12 (t = 3.27), 13 (t = 6.29) and between DSS and DSS+RSV groups at day 6 (t = 3.00) (b). Significant differences between sexes were observed in DSS groups on day 13 (t = 3.07) and in DSS+RSV groups on days 6 (t = 4.13), 8 (t = 4.34), 9 (t = 3.83) (d and e). CTRL, control; DSS, dextran sulfate sodium; RSV, resveratrol.

Variation showed these results:

**F values**	**a**	**b**	**c**	**d**	**e**
Interaction	6.77	4.40	1.74	2.34	3.81
Time	14.5	13.5	1.41	23.2	31.2
Column Factor	25.8	10.7	6.40	0.00967	11.0

Subjects (matching)	4.43	2.37	1.81	3.53	3.97

Figure 3. Discomfort time course. In females, there was a significant difference between CTRL and DSS groups at day 8 (t = 3.03), between CTRL and DSS+RSV groups at days 9 (t = 4.57), 10 (t = 4.26), 11 (t = 4.87), 12 (t = 5.48), 13 (t = 5.48) and between DSS and DSS+RSV groups at days 11 (t = 3.91), 12 (t = 3.81), 13 (t = 3.81) (a). In males, there was a significant difference between CTRL and DSS+RSV groups at day 6 (t = 3.14) (b). Significant differences between sexes were observed in DSS groups on days 11 (t = 3.56), 12 (3.56), 13 (t = 3.56); in DSS groups at day (t = 2.97) and in DSS+RSV groups on days 6 (t = 3.11), 8 (t = 2.99) (c, d and e). CTRL, control; DSS, dextran sulfate sodium; RSV, resveratrol.

Variation showed these results:

**F values**	**a**	**b**	**c**	**d**	**e**
Interaction	4.29	1.12	2.28	0.977	2.15
Time	10.7	11.8	2.28	8.86	21.0
Column Factor	8.16	3.78	22.1	5.61	2.24
Subjects (matching)	5.14	4.17	2.00	4.17	5.83
